# Supplementary material for: Potential Barriers to the Implementation of Computer-Based Simulation in Pharmacy Education: A Systematic Review
Source: Pharmacy (Basel). 2023 May 17;11(3):86. doi: 10.3390/pharmacy11030086 (PMC10204401; doi:10.3390/pharmacy11030086)
Supplement: Supplementary file 1 [file pharmacy-11-00086-s001.zip › PRISMA Checklist_pharmacy-2313901.pdf]

PRISMA Checklist

While the PRISMA checklist was initially developed for systematic reviews and meta-analyses, it can be adapted for use in systematic narrative reviews with some justifications. The underlying principles of transparency, rigor, and comprehensiveness in conducting and reporting reviews apply to both types of reviews, making the PRISMA checklist a valuable tool for ensuring high-quality reporting in systematic narrative reviews.

Section/topic	#	Checklist item	Information reported		Line number(s)
			Yes	No	
**ADMINISTRATIVE INFORMATION**					
**Title**					
Identification	1a	Identify the document as a protocol of a systematic narrative review	Yes	☐	2 - 3
Update	1b	If the protocol is for an update of a previous systematic review, identify as such	☐	No	N/A
**Authors**					
Contact	2a	Provide name, institutional affiliation, and e-mail address of all protocol authors; provide physical mailing address of corresponding author	Yes	☐	4 - 8
Contributions	2b	Describe contributions of protocol authors and identify the guarantor of the review	Yes	☐	451-457
**Abstract**	3	Provide a summary to the project	Yes	☐	9-32
**Amendments**	4	If the protocol represents an amendment of a previously completed or published protocol, identify as such and list changes; otherwise, state plan for documenting important protocol amendments	☐	☐	N/A
**Support**					
Sources	5a	Indicate sources of financial or other support for the review	Yes	☐	458-459
Sponsor	5b	Provide name for the review funder and/or sponsor	☐	☐	N/A
Role of sponsor/funder	5c	Describe roles of funder(s), sponsor(s), and/or institution(s), if any, in developing the protocol	☐	☐	N/A
**INTRODUCTION**					
**Rationale**	6	Describe the rationale for the review in the context of what is already known	Yes	☐	38-73
**Objectives**	7	Provide an explicit statement of the question(s) the review will address with reference to participants, interventions, comparators, and outcomes (PICO)	Yes	☐	73-78

Section/topic	#	Checklist item	Information reported		Line number(s)
			Yes	No	
**METHODS**					
Protocol registration	8	If registered, provide the name of the registry (e.g., PROSPERO) and registration number in the Abstract	☐	No	N/A
Eligibility criteria	9	Specify the study characteristics (e.g., PICO, study design, setting, time frame) and report characteristics (e.g., years considered, language, publication status) to be used as criteria for eligibility for the review	Yes	☐	110-118
Information sources	10	Describe all intended information sources (e.g., electronic databases, contact with study authors, trial registers, or other grey literature sources) with planned dates of coverage	Yes	☐	88-96
Search strategy	11	Present draft of search strategy to be used for at least one electronic database, including planned limits, such that it could be repeated	Yes	☐	98-106
Selection process	12	State the process that will be used for selecting studies (e.g., two independent reviewers) through each phase of the review (i.e., screening, eligibility, and inclusion in meta-analysis)	Yes	☐	107-109
Data collection/extraction process	13	Describe planned method of extracting data from reports (e.g., piloting forms, done independently, in duplicate), any processes for obtaining and confirming data from investigators	Yes	☐	120-132+ Supplementary table 1 & 2
Data items	14	List and define all variables for which data will be sought (e.g., PICO items, funding sources), any pre-planned data assumptions and simplifications	Yes	☐	133-141
Quality appraisal of included studies (if applicable)	15	evaluating the methodological quality, validity, and potential biases in each study included in the review.	☐	No	N/A
Narrative synthesis approach	16	List and define all outcomes for which data will be sought, including prioritization of main and additional outcomes, with rationale	Yes	☐	142-179
**Results**					
Study selection	17	the process and outcomes of identifying, screening, and selecting studies for inclusion in the review.	Yes	☐	181-186 + Figure 1
Study characteristics	18	a summary of the key features of the studies included in the review.	Yes	☐	189-205
Quality appraisal of included studies (if applicable)	19	evaluating the methodological quality, validity, and potential biases in each study included in the review.	☐	No	N/A

Section/topic	#	Checklist item	Information reported		Line number(s)
			Yes	No	
Narrative synthesis of results	20	findings from the included studies are synthesized, analyzed, and discussed to address the research question(s) or objective(s) of the review.	Yes	☐	208-312
**Discussion**					
Summary of evidence	21	an overview of the main findings from the review, highlighting the most important and relevant evidence that addresses the research question(s) or objective(s).	Yes	☐	313-364
Implications for practice, policy, and research	22	bridge the gap between research and practice, providing recommendations and insights that can be applied by practitioners, educators, policymakers, and other stakeholders in the field.	Yes	☐	365-413
Limitations	23	Potential weaknesses, shortcomings, or biases in your review.	Yes	☐	414-436
**Conclusion**					
Conclusion	24	a concise and coherent summary of your main findings, address the research question(s) or objective(s), and emphasize the significance and implications of your review.	Yes	☐	437-446

References:

Liberati A., Altman D.G., Tetzlaff J., et al. The PRISMA statement for reporting systematic reviews and meta-analyses of studies that evaluate healthcare interventions: explanation and elaboration. BMJ. 2009;339:b2700.

Moher D., Liberati A., Tetzlaff J., Altman D.G.; PRISMA Group. Preferred reporting items for systematic reviews and meta-analyses: the PRISMA statement. PLoS Med. 2009;6(7):e1000097.

Popay J., Roberts H., Sowden A., et al. Guidance on the conduct of narrative synthesis in systematic reviews: a product from the ESRC Methods Programme. Lancaster: Institute of Health Research; 2006.

Green B.N., Johnson C.D., Adams A. Writing narrative literature reviews for peer-reviewed journals: secrets of the trade. J Chiropr Med. 2006;5(3):101–17.
