# Supplementary material for: Potential Barriers to the Implementation of Computer-Based Simulation in Pharmacy Education: A Systematic Review
Source: Pharmacy (Basel). 2023 May 17;11(3):86. doi: 10.3390/pharmacy11030086 (PMC10204401; doi:10.3390/pharmacy11030086)
Supplement: Supplementary file 1 [file pharmacy-11-00086-s001.zip › Supplementary material_pharmacy -2313901.pdf]

Supplementary Information

Table S1. Search strategy

Serial No.	Database	Search String	Date of Search	Results
1	*Education Source*	*(computer-based simulation OR pharmacy practice education OR pharmacy education OR online simulation OR virtual simulation OR virtual patient OR serious gaming OR case-based simulation OR standardised patient) AND (DE "Pharmacy Education" OR DE "Simulation" OR DE "Computer Uses in Education" OR DE "Distance Education" OR DE "E-Learning" OR DE "Educational Games" OR DE "Teaching Methods")*	*4th March 2022*	*487*
2	*Ovid EMBASE*	*((computer-based simulation.mp. OR computer simulation.mp. OR online simulation.mp. OR virtual simulation.mp. OR virtual patient.mp. OR serious gaming.mp. OR case-based simulation.mp. OR standardized patient.mp.) AND (pharmacy practice education.mp. OR pharmacy education.mp.))*<br><br>*Note: ".mp." stands for "multi-purpose" and searches for terms in multiple fields (e.g. title, abstract, keywords, etc.).*	*4th March 2022*	*353*
3	*ERIC*	*(((("computer-based simulation" OR "online simulation" OR "virtual simulation" OR "serious gaming" OR "case-based simulation" OR "standardized patient") AND ("pharmacy practice education" OR "pharmacy education")) OR "virtual patient") OR "simulated patient") Full Text*	*4th March 2022*	*261*
4	*CINAHL*	*Search Alert: ((("computer-based simulation"[Title/Abstract] OR "pharmacy practice education"[Title/Abstract] OR "pharmacy education"[Title/Abstract] OR "online simulation"[Title/Abstract] OR "virtual simulation"[Title/Abstract] OR "virtual patient"[Title/Abstract] OR "serious gaming"[Title/Abstract] OR "case-based simulation"[Title/Abstract] OR "standardised patient"[Title/Abstract])) OR (("computer simulation"[MeSH Terms] OR "computer simulation"[Title/Abstract] OR "simulation training"[Title/Abstract] OR "virtual learning"[Title/Abstract] OR "gamification"[Title/Abstract] OR "serious gaming"[MeSH Terms] OR "serious gaming"[Title/Abstract]) AND ("pharmacy education"[MeSH Terms] OR "pharmacy education"[Title/Abstract] OR "pharmacy practice"[MeSH Terms] OR "pharmacy practice"[Title/Abstract]))) AND ("education, pharmacy"[MeSH Terms] OR "education, pharmacy"[Title/Abstract]) Full Text; Published Date: 20000101-20221231 AND Apply equivalent subjects on 2022-03-15 07:28 AM"*	*4th March 2022*	*210 results*

5	*Ovid Medline*	*ALL (("Computer Simulation"[Mesh] OR "Models, Educational"[Mesh] OR "Education, Pharmacy"[Mesh] OR "Computer-Assisted Instruction"[Mesh] OR "Teaching"[Mesh]) AND ("Education, Pharmacy"[Mesh] OR "Education, Distance"[Mesh] OR "Patient Simulation"[Mesh] OR "Patient-Centered Care"[Mesh] OR "Problem-Based Learning"[Mesh] OR "Standardized Patients"[Mesh] OR "Video Games"[Mesh] OR "Virtual Reality Exposure Therapy"[Mesh] OR "Virtual Reality"[Mesh])) OR ("Computer Simulation"[tiab] OR "Models, Educational"[tiab] OR "Pharmacy Education"[tiab] OR "Computer simulation"[tiab] OR "Teaching"[tiab] OR "Online Simulation"[tiab] OR "Virtual Simulation"[tiab] OR "Virtual Patient"[tiab] OR "Serious Gaming"[tiab] OR "Case-Based Simulation"[tiab] OR "Standardised Patient"[tiab]) AND (LIMIT-TO (LANGUAGE , "English"))*	*4th March 2022*	*189 results*

Table S2. Characteristics of the included studies

**Study (Authors and year)**	**Country**	**Study Focus**	**Data collection methods/ Evaluation methods**	**Participants**	**Computer-based simulation**	**Factors identified as barriers**<br><br>(Cultural, Process, and Academic)
Al-Dahir, Bryant, Kennedy, & Robinson, 2014 [65]	USA	To evaluate computer simulator as a learning method	Knowledge-based pre-post-MCQs.<br><br>Evaluative survey - Likert scale	Pharmacy undergraduates at one university	DecisionSim (Formerly vpSim)	**Cultural barriers**<br><br>Students' attitude/resistance to change [6,12,65]<br><br>**Process barriers**<br><br>1) Time needed by students to complete the exercise [65,67]<br><br>2) extensive case and simulation development time [6,12,66,67,69]<br><br>3) Students and Educators technical support (connectivity) [6,65]
Neal Benedict, 2010 [66]	USA	Incorporating effective and active-learning strategies	Knowledge-based questions.<br><br>SOAP (Subjective-objective assessment-plan).<br><br>Post course evaluative survey.	Pharmacy undergraduates at one university		
Benedict, Schonder, & McGee, 2013 [12]	USA	To assess the effectiveness of computer simulation	Pre and post knowledge quiz<br><br>Final exam scores.	Pharmacy undergraduates at one university		

		and promote self-directed learning	Post-course evaluative survey (Likert scale).			4) (organisation) cost [6,68,69]<br>5) Staff training [6]<br>**<u>Academic barriers</u>**<br>1) Curriculum constraints<br>Matching the course aim and needs [65]<br>2) Motivating and engaging students' needs and expectations [12,68].
Lichvar et al., 2016 [67]	USA	To design and evaluate the integration of a virtual patient activity.<br>And study an effective active-learning strategy.	Pre and post knowledge quiz<br>End-of-year exam (to assess knowledge retention)<br>Evaluative survey (Likert scale and open-ended questions)	Pharmacy undergraduates at one university		
Olin & Cole, 2015 [68]	USA	To describe computer-based clinical simulation cases (CBCSCs) and review student perceptions of this learning strategy	Evaluative survey (Likert scale)	Pharmacy undergraduates at two campuses		
Michael A. Smith, Mohammad, & Benedict, 2014 [6]	USA	To assess student satisfaction and learning of course objectives	The post-course survey, and a pre-simulation and post-simulation tests were used to assess student learning	Pharmacy undergraduates at one university		
Michael A Smith, Siemianowski, & Benedict, 2016 [69]	USA	To expand the use of virtual patients by sharing case scenarios across two schools	Pre and post-tests assessed student learning, data collected regarding the development and sharing of cases.<br>The survey was conducted to measure faculty perception and student satisfaction	Pharmacy undergraduates and 3 educators at two schools		
Ambroziak, Ibrahim, Marshall, & Kelling, 2018 [7]	USA	To evaluate a computer simulator and assess the dispensing knowledge	Post-course survey (with open and closed-ended questions)	Pharmacy undergraduates at one university	MyDispense	<u>Cultural barriers</u><br>Students' attitude/resistance to change [7,71,72]

Ferrone, Kebodeaux, Fitzgerald, & Holle, 2017 [70]	USA	To evaluate computer simulator and assess the dispensing knowledge and student perception	Post-simulation survey (Likert scale and open-ended questions)	Pharmacy undergraduates at three Universities		<u>Process barriers</u><br><br>1) Time needed by students to complete the exercise [4,71,72]<br><br>2) extensive case and simulation development time [4,7,28,72]<br><br>3) Software use and updates [4,7,28,70,71]<br><br>4) Lack of a clear process and procedures and/or faculty expertise [70]<br><br><u>Academic barriers</u><br><br>1) Curriculum constraints (Matching the course aim and needs) [71]<br><br>2) Motivating and engaging students<br><br>(Students' needs and expectations) [72]<br><br>3) Lack of a clear process and procedures and/or faculty expertise[70]
Mak, Fitzgerald, Holle, Vordenberg, & Kebodeaux, 2021 [4]	Australia	To describes the use of a virtual simulation in enhancing student learning	Commentary on "MyDispense" in the didactic and experiential settings to meet educational outcomes and the challenges of virtual simulation	Pharmacy undergraduates		
McDowell et al., 2016 [28]	Australia	To develop and evaluate a computer simulator and assess student knowledge	Analysis of student examination results and post-course survey	Pharmacy undergraduates at one university		
Shin, Tabatabai, Boscardin, Ferrone, & Brock, 2018 [71]	USA	To demonstrate the feasibility of integrating computer simulation and assess student perceptions	pre- and post-tests in class and three surveys (for each phase and overall experience)	Pharmacy undergraduates at one university		
Tai et a;., 2020 [72]	USA	Assess student perception and knowledge in introductory pharmacy practice experiences	Post-course surveys were conducted by students.<br><br>Also, preceptors were asked to complete a survey	Pharmacy undergraduates and preceptors at one university		
Richardson et al.,, 2019 [93]	different European countries	To develop and show proof of concept of the computer-based simulation	A blend of formal meetings, distance evaluation (using formal forms and email feedback)<br><br>Evaluation forms were a mixture of quantitative and qualitative questions	A steering group of European pharmacists provided data for computer simulation development	Keele virtual patient (Keele University)	<u>**Cultural barriers**</u><br><br>Educators' attitude/resistance to change [93]<br><br><u>**Process barriers**</u><br><br>1) Students and Educators

Thompson et al., 2020 [8]	UK	To evaluate perceptions on the integration, of computer simulator	Telephone interviews (Semi-structured) to discuss the students’ perspectives on the use of the virtual patient or non-interactive case studies	Pharmacy undergraduate (pre-registration) at one university		technical support [8]<br><br>2) Software use and updates [8,42,84,93]<br><br>3) Lack of a clear process and procedures and/or faculty expertise [8]<br><br>**Academic barriers**<br><br>1) Curriculum constraints (Matching the course aim and needs) [42,93]<br><br>2) Motivating and engaging students<br><br>(Students’ needs and expectations) [8,42,84]
Thompson et al., 2020 [42]	UK	To evaluate virtual patient (VP) and non-interactive (NI) case studies, concerning knowledge, skill, and confidence development	Pre- and post-knowledge quiz. questionnaire (Likert scale and open-ended questions)	Pharmacy students (preregistered) at one university		
Bracegirdle & Chapman, 2010 [84]	UK	To demonstrate the computer simulator design and report the integration experience	Collective feedback	Pharmacy undergraduates at one university		
Loke et al., 2010[86]	New Zealand	To evaluate the learning method using a computer simulator and students’ perception	Audio recordings and observations in class Focus groups	Pharmacy undergraduates at one university	SimPharm	**Cultural barriers**<br><br>Educator resistance to change [86]<br><br>**Process barriers**<br><br>1) Time needed by students to complete the exercise [86]<br><br>2) Software use and updates [87]<br><br>**Academic barriers**<br><br>- Curriculum constraints (Matching the course aim and needs) [87]
Wright et al., 2020[87]	New Zealand	To evaluate the knowledge and skills delivered by the computer simulator	A pre-and post-intervention crossover study design	Practising pharmacists		

Bravo et al., 019 [88]	Canada	To assess students' perceptions using a computer simulation	a semi-structured interview conducted by VIC's project team members.<br><br>The interviews were audio-recorded, transcribed, and coded for extracting themes	Pharmacy undergraduates from one university	Virtual Interactive Case System	**<u>Cultural barriers</u>**<br><br>Students' attitude/resistance to change [89]<br><br>**<u>Process barriers</u>**<br><br>1) Time needed by students to complete the exercise [88]<br><br>2) Students and Educators technical support [88]<br><br>3) Lack of a clear process and procedures and/or faculty expertise [88,89]<br><br>**<u>Academic barriers</u>**<br><br>1) Curriculum constraints<br><br>(Matching the course aim and needs) [88]<br><br>2) Motivating and engaging students<br><br>(Students' needs and expectations) [88,89]
Dahri et al., 2019[89]	Canada	To investigate students' perceptions towards computer simulation	Post-course survey and focus group participation	Pharmacy undergraduates at one university		
Bindoff et al.,2014 [82]	Australia	To present and evaluate the computer simulator's effectiveness and actively engaging learning experience	Pre and post knowledge quiz<br><br>Evaluative survey<br><br>(Likert scale and open-ended questions)	Pharmacy undergraduates from one university	Pharmacy Simulator	**<u>Cultural barriers</u>**<br><br>1) Educators' attitude/resistance to change [82]<br><br>2) Students' attitude/resistance to change [82]<br><br>**<u>Process barriers</u>**<br><br>1) Software use and updates<br><br>Design limitation [82,83]<br><br>2) Lack of a clear process and procedures and/or faculty expertise [83]
Tait et al.,2018 [83]	Australia	To investigate perception and experience using computer simulation	A post-simulation questionnaire	Master of Pharmacy students at one university		

						**Academic barriers**<br><br>1) Curriculum constraints (Matching the course aim and needs) [82,83]<br><br>2) Motivating and engaging students (Students needs and expectations) [82]
Zary, Johnson, Boberg, & Fors, 2006 [9]	Sweden	To evaluate a computer simulator	Post-course surveys and on-site observations were performed when possible.<br><br>(Likert scale and free text comments)	Medicine, dental and pharmacy undergraduates	Web-based Simulation of Patients (Web-SP)	**Cultural barriers**<br><br>Students' attitude/resistance to change [89].<br><br>**Process barriers**<br><br>1) Extensive case and simulation development time [9]<br><br>2) Software use and updates/Design limitation [9]<br><br>3) Lack of a clear process and procedures and/or faculty expertise [9]<br><br>4) Cost [9]<br><br>**Academic barriers**<br><br>Curriculum constraints (Matching the course aim and needs) [88]
Marriott, 2007 [3]	Australia	To assess and evaluate a computer simulator's use as a learning tool	Post-assessment survey) to determine the program's ease of use and its perceived value	Pharmacy undergraduates at one School/University	A purpose-designed computer program	**Cultural barriers**<br><br>Students' attitude/resistance to change [89]<br><br>**Process barriers**<br><br>1) Extensive case and simulation development time [9]<br><br>2) Software use and updates/Design limitation [9]
Marriott, 2007 [79]	Australia	To assess and evaluate a computer simulator's use as a learning tool	Review of the software design, features, and functions	Pharmacy undergraduates at one School/University		

						3) Lack of a clear process and procedures and/or faculty expertise [9,79]<br><br>4) Cost [9]<br><br>**<u>Academic barriers</u>**<br><br>- Curriculum constraints (matching the course aim and needs) [88]
Caylor, Aebersold, Lapham, & Carlson, 2015 [73]	USA	to examine the use and effectiveness of computer simulation in multi-professional learning	Pre and post IPE<br><br>Perception Scale (Likert scale)<br><br>Pre and post teamwork<br><br>Questionnaire (Likert scale)<br><br>Team Performance<br><br>Observation Tool – Faculty members<br><br>Post-course evaluative survey.<br><br>(Likert scale and open-ended Q's)	Undergraduates in nursing, medicine, and pharmacy at same University	Second Life	**<u>Process barriers</u>**<br><br>1) extensive case and simulation development time [73]<br><br>2) Software use and updates/Design limitation Technical issues – awkward navigation [73]<br><br>3) Unclear process and procedures [73]<br><br>4) Cost (organisation) [73]<br><br>**<u>Academic barriers</u>**<br><br>Curriculum constraints<br><br>(Matching the course aim and needs (matching the need for interdisciplinary requirements))[73]
Benedict & Schonder, 2011[39]	USA	To implement and assess the effectiveness of a computer simulation in teaching	Pre and post knowledge quiz<br><br>Final exam scores<br><br>Post-course evaluative survey (Likert scale)	Pharmacy undergraduates from one university	PharmaCAL	**<u>Process barriers</u>**<br><br>1) Extensive case and simulation development time [39]<br><br>2) Software use and updates [39]<br><br>4) Cost [39]<br><br>**<u>Academic barriers</u>**

						- Motivating and engaging students (students' needs and expectations) [39]
Chaikoolvatana & Goodyer, 2003 [85]	UK	To validate a computer simulator design and its use for learning	Care plan developed by students<br><br>Face-to-face consultation<br><br>Post-course evaluative survey (Likert scale)	Pharmacy undergraduates at one university	Multimedia case history program (MCHP)	**<u>Process barriers</u>**<br><br>1) extensive case and simulation development time [85]<br><br>2) Software use and updates - Design limitation [85]
Cláudio et al., 2015 [97]	Portugal	To present and evaluate computer simulation as a learning tool	Evaluative survey (Likert scale and open-ended questions)	Seven qualified pharmacists	Virtual Human	**<u>Cultural barriers</u>**<br><br>1) Educators' attitude/resistance to change (cannot fully replace students' assessment traditional method) [97]<br><br>2) Students' attitude/resistance to change [97]<br><br>**<u>Process barriers</u>**<br><br>1) extensive case and simulation development time [97]<br><br>2) Software use and updates - Design limitation [97]<br><br>**<u>Academic barriers</u>**<br><br>1) Motivating and engaging students<br><br>(Students' needs and expectations) [97]<br><br>2) Curriculum constraints(Matching the course aim and needs, as it lacks active listening or emphatic capacity in a true community pharmacy environment) [97]

Ma, Finn, Czosnowski, Whitman, & Cawley, 2011 [5]	USA	To assess the impact of the computer simulation on student knowledge during another mannequin-based exercise	Post simulation survey with questions included demographic information, computer simulation scores, and satisfaction-related questions	Pharmacy undergraduates at one university	MicroSim	**<u>Cultural barriers</u>**<br>Students' attitude/resistance to change [5]<br>**<u>Process barriers</u>**<br>1) extensive case and simulation development time [5]<br>2) Software use and updates/Design limitation [5]<br>3) Cost [5]<br>**<u>Academic barriers</u>**<br>Curriculum constraints (Matching the course aim and needs) [5]
Douglass, Casale, Skirvin, & DiVall, 2013 [74]	USA	To implement and assess the impact of computer simulation on students' knowledge and skills	Pre and post-test for evaluating clinical competence.<br>Post-course evaluative survey.(Likert scale)	Pharmacy undergraduates at one university	TheraSim	**<u>Cultural barriers</u>**<br>Students' attitude/resistance to change [74]<br>**<u>Process barriers</u>**<br>1) extensive case and simulation development time [74]<br>2) Software use and updates/Design limitation [74]<br>3) Cost (organisation) [74]
Hussein & Kawahara, 2006 [75]	USA	To promote active learning and knowledge	Knowledge assessed based on<br>1) the care plan and messages left on the telephone<br>2) Final knowledge exam	Pharmacy undergraduates and six faculty members at one university	Computer telephony interactive voice response system (IVR) and a text-to-	**<u>Cultural barriers</u>**<br>Students' attitude/resistance to change [75]<br>**<u>Process barriers</u>**<br>1) Extensive case and simulation development time [75]

			Educators’ evaluative survey Likert scale)<br><br>Students’ evaluative survey (Likert scale and open-ended questions)		speech (TTS) system	2) Software use and updates/Design limitation [75]<br><br>3) Cost (organisation) [75]<br><br>**Academic barriers**<br><br>1) Motivating and engaging students [75]<br><br>2) Lack of a clear process and procedures and/or faculty expertise [75]
Fens, Dantuma- Wering, & Taxis, 2020 [91]	Netherland s	To present and evaluate a computer simulation and assess a wide range of competencies	Students are assessed individually and as a pharmacy team.<br><br>(The pharmacy team, with the largest number of patients, wins the game)	Postgraduate pharmacy students at three universities (Groningen, Utrecht, and Leiden)	Pharmacy Game<br><br>Previously known as “GIMMICS/ P harmG”	**Process barriers**<br><br>Software use and updates/Design limitations (Different programs needed to run the simulation) [91].<br><br>Utilising Microsoft applications that allow sharing documents, audio, and video calls (Human to human) interaction
Lim et al., 2020 [92]	Australia & Malaysia	To assess knowledge and perceptions among students using a computer simulator	Self-administered questionnaire and focus group	Pharmacy undergraduates at two campuses.	The Monash OSCE Virtual Experience (MOVE)	**Cultural barriers**<br><br>1) Scepticism [92].<br><br>2) Students’ attitude/resistance to change [92]<br><br>**Process barriers**<br><br>1) Software use/access [92]<br><br>2) Lack of a clear process and procedures (usability and training issues) [92]<br><br>**Academic barriers**<br><br>1) Motivating and engaging students [92]<br><br>2) Curriculum constraints [92]

J. Marriott, Styles, & McDowell, 2012 [80]	Australia	To present a computer simulator and the integration experience within teaching the curriculum	Post-course students' feedback survey for two consecutive years.<br><br>A student focus group discussion was conducted.<br><br>A comprehensive evaluation was undertaken of staff and student use of Pharmville	Pharmacy undergraduates	Pharmville	**<u>Process barriers</u>**<br><br>1) extensive case and simulation development time [80]<br><br>2) Software use and updates/Design limitation [80]<br><br>**<u>Academic barriers</u>**<br><br>1) Motivating and engaging students<br><br>2) Curriculum constraints - implement Pharmville activities (course design) [80]<br><br>**-** Need to meet accreditation standards [80]<br><br>3) Lack of faculty expertise [80].
Menendez et al., 2015 [90]	Brazil	To present and evaluate computer simulation in teaching	Post-course surveys and on-site observations were performed when possible	Pharmacy undergraduates at one university	PharmaVP	**<u>Cultural barriers</u>**<br><br>(Scepticism) students' attitude/resistance to change [90]<br><br>**<u>Process barriers</u>**<br><br>1) Software use and updates/Design limitation [90]<br><br>**<u>Academic barriers</u>**<br><br>1) Motivating and engaging students [90]
Park & Summons, 2013 [81]	Australia	To present a computer simulator and students' perceptions	Evaluative survey – Likert scale	Pharmacy undergraduates at three universities	The Virtual Pharmacy Patient	**<u>Process barriers</u>**<br><br>Software use and updates/Design limitation [81]<br><br>**<u>Academic barriers</u>**<br><br>Motivating and engaging students [81]

Taglieri, Crosby, Zimmerman, Schneider, & Patel, 2017 [76]	USA	To assess the effect of incorporating Computer simulation on student competence and confidence	Pre-survey, interim survey, post-survey (Likert scale and open-ended questions)<br><br>Performance in mock clinic	Pharmacy undergraduates at one university	Shadow Health Digital Clinical Experience (DCE)	**<u>Cultural barriers</u>**<br><br>Students' attitude/resistance to change [76]<br><br>**<u>Process barriers</u>**<br><br>1) Software use and updates/Design limitation [76]<br><br>2) Cost (organisation) [76]<br><br>**<u>Academic barriers</u>**<br><br>1) Curriculum constraints<br><br>(Course Accreditation/course needs and aims) [76]<br><br>2) Motivating and engaging students [76]
Villaume, Berger, & Barker, 2006 [77]	USA	To assess students' knowledge using computer simulation	Post assignment survey was conducted to collect feedback regarding students' experiences with the tool.<br><br>Knowledge test (by a number of MCQs as part of the final examination to examine students' knowledge retention and efficiency of incorporating the tool. Students' script for VP - graded final exam – Knowledge retention.	Pharmacy undergraduates	The Auburn University Virtual Patient (AUVP) simulation	**<u>Cultural barriers</u>**<br><br>Students' attitude/resistance to change[77]<br><br>**<u>Process barriers</u>**<br><br>1) extensive simulation development time and knowledge [77]<br><br>2) Software use and updates/Design limitation [77]<br><br>**<u>Academic barriers</u>**<br><br>1) Motivating and engaging students [77]<br><br>2) Curriculum constraints [77]<br><br>3) Lack of faculty expertise [77]

Battaglia, Kieser, Bruskiewitz, Pitterle, & Thorpe, 2012 [78]	USA	To present the development, implementation, and assess the effectiveness of computer simulation	Pre and post assessment (Likert scale, multiple choice questions (MCQs) and short answer questions)	Pharmacists (42) and pharmacy students at one university	Virtual patient training via the online Moodle platform	**Process barriers**<br>1) Software use and updates/Design limitation [78]<br>2) Usability of the software (access and procedures) [78]<br>**Academic barriers**<br>1) Motivating and engaging students[78]<br>2) Lack of faculty expertise [78]
Cavaco & Madeira, 2012 [35]	Europe	To describe how virtual patients are being used to simulate real-life clinical scenarios in undergraduate pharmacy education in Europe	Exploratory cross-sectional survey regarding the use of computer-based simulations,<br>perceptions of computer-based simulations in education (Likert scale)	Pharmacy undergraduates at European Pharmacy<br>Students Association (EPSA) Annual Congress, 2011 (23 countries and 46 Universities)	The different computer-based virtual simulations used across Europe<br>(Depends on students' individual encounter/experience)	**Cultural barriers**<br>1) Lack of collaboration bet. Local market and educational institute [35]<br>2) Resistance to change (organisation) [35]<br>3) Resistance to change (Educators) [35]<br>**Process barriers**<br>1) Software use and updates/Design limitation [35]<br>2) Cost (organisation) [35]<br>3) Lack of a clear process and procedures [35]<br>**Academic barriers**<br>1) Curriculum constraints<br>(Course Accreditation/course needs and aims) [35]<br>2) Motivating and engaging students [35]

TableS3. Characteristics of the included grey literature (AACODS)

(Authors, publication year, study title)	Authority	Accuracy	Coverage	Objectivity	Date	Significance	Comments on Implementing computer-based, virtual simulation in pharmacy education
Reports							
(Accreditation Council for Pharmacy Education, 2015) [2]	Accreditation Council for Pharmacy Education	High-quality education standard.<br><br>Accurate consideration of the expansion of pharmacy education scope<br><br>Detailed updates:<br><br>Educational outcomes,<br><br>Structure<br><br>Procedures<br><br>Assessment of standards<br><br>Reports from other bodies and institutes “e.g., IOM, AACP, JCPP, AACN”<br><br>Previous feedback from ACPE stakeholders<br><br>Updates regarding (state laws and	Standards in quality pharmacy education<br><br>Continuous updating and guidance to best practices	Changes and challenges in pharmacy education<br><br>Discussing the needs of different stakeholders	Standards for the year 2016 (which is the most recent report from ACPE)<br><br>This document is regularly revised and forms the basis for the most updated versions (version 2) of rubrics used till this year 2021 in accreditation visits	Insight into educational needs.<br><br>Different stakeholders stand<br><br>It directly affects policy-making decisions.<br><br>Guides the decision makers’ priorities in curriculum standards	Students should have (The knowledge, skills, and abilities to provide patient-centred care and solve problems)<br><br>Emphasis on introductory pharmacy practice experiences (IPPE)<br><br>Simulations are not intended to replace direct-patient experiences<br><br>Simulated practice experiences (a maximum of 60 clock hours of the total 300 hours)<br><br>Simulation hours do not substitute for the required IPPE time<br><br>Importance of availability of dedicated resources (faculty)<br><br>Relevance to course aim and objectives

		expansion of pharmacy role and scope)					
(Accreditation Council for Pharmacy Education, 2020)<br><br>[18]	Accreditation Council for Pharmacy Education	Clarify the standards<br><br>Explain the accreditation process for ACPE<br><br>Explains the role of simulation in accredited programs	Accurate coverage of the report aims<br><br>Highlighting the role of different key factors within the accreditation process	Various updates and feedback (From different stakeholders)<br><br>The accreditation process step by step<br><br>Standards and procedures for implementing simulation	Up-to-date standards and procedure reports, completed in January 2021<br><br>Mainly referencing the accreditation standards in the ACPE standards report 2016	Regulates the relationship between ACPE and the pharmacy program of a given institute<br><br>ACPE standards ensure high education standards<br><br>Keep stakeholders up to date on the most recent challenges	Acceptable and non-acceptable activities for pharmacy simulation set by ACPE<br><br>Role of simulation to complement traditional training methods<br><br>Standards for using and integrating simulation into pharmacy curriculum
(Accreditation Standards for Pharmacy Programs in Australia and New Zealand, 2020)<br><br>[64]	The Australian Pharmacy Council (APC)	Safe and socially accountable practice<br><br>Governance and quality<br><br>Program (Design, implementation, and resourcing)<br><br>Student/intern experience<br><br>Outcomes and assessment	assure the quality of pharmacy education programs and promote further improvement in their quality.	Used as the basis for the accreditation of degree programs in both Australia and New Zealand<br><br>N.B. separate Accreditation Standards exist for intern training programs (ITP) in Australia and New Zealand	Up-to-date standards and procedure reports, effective from 1 January 2020	Ensure that graduates of degree programs and applicants for initial general registration meet all of the competencies required for practice under supervision as an intern, and unsupervised practice as a pharmacist respectively	There is no requirement for engagement in real environments (but highly desirable), however, providers should describe how the simulated environments they use to prepare students/interns for real experience<br><br>(As seen in Criterion 3.8)<br><br>"simulation is not limited to interprofessional learning but was identified in a 2010 Health Workforce Australia report as a particularly useful approach where direct interprofessional experience is not readily available" (It is also an expectation of the Pharmacy Action Plan in New Zealand)

(Raehl, C.L., Baldwin, J.N., Carter, R.A., Crabtree, B.L., Yanchick, V.A., Maine, L.L., 2013)<br><br>[63]	The American Association of Colleges of Pharmacy Argus Commission	This report aims to<br><br>Identify five sectors that would affect pharmacy education in future<br><br>Discuss expected changes to pharmacy education	Game changers in pharmacy education<br><br>(It doesn't enforce any policy change but rather discusses challenges and offers recommendations)	Carefully discussed<br><br>Logically analysed<br><br>Set of recommendations	Clearly stated the date (2012-2013) and a detailed list of the used references	High-ranked decision makers involved<br><br>Expected change in pharmacy education<br><br>Systematic analysis of the presented ideas<br><br>Set of recommendations that is thought to help AACP members to decide their policies	Active engagement with different stakeholders<br><br>Promote a joint independent entity that combines all stakeholders to facilitate collaboration<br><br>The proposed policy statements and set of recommendations:<br><br>In Policy statement #1,<br><br>a) AACP encourages meeting stakeholders' needs and engaging them in decision-making,<br><br>b) link academia to market needs and<br><br>c) explore education style preferences among students<br><br>In policy statement #2,<br><br>AACP encourages its members to adopt and adapt to new changes (flexible and supportive culture)<br><br>-In policy statement #3,<br><br>AACP encourages its members to study the best adoption strategy to ensure the successful utilisation of new technology
